# MOSAIC (MOthers' Advocates In the Community): protocol and sample description of a cluster randomised trial of mentor mother support to reduce intimate partner violence among pregnant or recent mothers

**DOI:** 10.1186/1471-2458-9-159

**Published:** 2009-05-27

**Authors:** Angela J Taft, Rhonda Small, Kelsey L Hegarty, Judith Lumley, Lyndsey F Watson, Lisa Gold

**Affiliations:** 1 Mother and Child Health Research, La Trobe University, 324-328 Little Lonsdale St, Melbourne, Vic 3000, Australia; 2 Department of General Practice, University of Melbourne, Melbourne, Victoria, Australia; 3 Deakin Health Economics, Deakin University, Melbourne, Victoria, Australia

## Abstract

**Background:**

Intimate partner violence (IPV) is prevalent globally, experienced by a significant minority of women in the early childbearing years and is harmful to the mental and physical health of women and children. There are very few studies with rigorous designs which have tested the effectiveness of IPV interventions to improve the health and wellbeing of abused women. Evidence for the separate benefit to victims of social support, advocacy and non-professional mentoring suggested that a combined model may reduce the levels of violence, the associated mental health damage and may increase a woman's health, safety and connection with her children. This paper describes the development, design and implementation of a trial of mentor mother support set in primary care, including baseline characteristics of participating women.

**Methods/Design:**

MOSAIC (MOtherS' Advocates In the Community) was a cluster randomised trial embedded in general practice and maternal and child health (MCH) nursing services in disadvantaged suburbs of Melbourne, Australia. Women who were pregnant or with infants, identified as abused or symptomatic of abuse, were referred by IPV-trained GPs and MCH nurses from 24 general practices and eight nurse teams from January 2006 to December 2007. Women in the intervention arm received up to 12 months support from trained and supported non-professional mentor mothers. Vietnamese health professionals also referred Vietnamese women to bilingual mentors in a sub-study. Baseline and follow-up surveys at 12 months measured IPV (CAS), depression (EPDS), general health (SF-36), social support (MOS-SF) and attachment to children (PSI-SF). Significant development and piloting occurred prior to trial commencement. Implementation interviews with MCH nurses, GPs and mentors assisted further refinement of the intervention. In-depth interviews with participants and mentors, and follow-up surveys of MCH nurses and GPs at trial conclusion will shed further light on MOSAIC's impact.

**Discussion:**

Despite significant challenges, MOSAIC will make an important contribution to the need for evidence of effective partner violence interventions, the role of non-professional mentors in partner violence support services and the need for more evaluation of effective health professional training and support in caring for abused women and children among their populations.

**Trial registration:**

ACTRN12607000010493

## Background

Intimate partner violence (IPV) has been defined as any behaviour within an intimate relationship that causes physical, psychological, or sexual harm. This includes:

• Physical aggression, such as hitting, kicking, and beating

• Psychological violence, such as intimidation, constant humiliation

• Forced intercourse and other sexual coercion

• Various controlling behaviours, such as isolation from family and friends, monitoring movements, financial control, and restricting access to services. [1]

It is experienced by a significant minority of women in the early childbearing years and is harmful to the mental and physical health of women and children [2]. Yet reviews of interventions to improve the health and wellbeing of abused women conclude that 'there is an urgent need for additional research using rigorous designs to test the effectiveness of IPV interventions for important clinical outcomes' [3,4].

Partner violence comprises a range of abusive behaviours, although its measurement and scope remain controversial issues [5]. In a 2005 national Australian population study, 15% of ever-partnered women reported physical or sexual abuse in a relationship. Just under half of the abused women (49%) reported that they had children at the time and 27% of their children had witnessed the abuse. Twelve percent of all women had themselves experienced sexual abuse as a child [6]. Thirty-six percent of abused women reported abuse when pregnant; 17% of these were abused for the first time when pregnant. Risk markers for partner violence among women consistently reflect disadvantage in studies across many countries. They include youth, early parity, low levels of education and low income [1,7,8].

### Abuse in pregnancy and after birth

Pregnancy is a time when violence against women by intimate partners (or others) can commence, continue or escalate [7]. Gazmararian and colleagues conducted a meta-analysis of studies with consistent measures of abuse and found that 4% to 8% of women were abused during pregnancy [9]. Partner violence can result in detrimental reproductive outcomes, including unwanted and unplanned pregnancies, poor birth outcomes, including low birth weight and pre-term birth, more pregnancy terminations than those among non-abused women and homicide of both mother and child [2,10].

### Children and abuse – resiliency and inter-generational effects

Partner and child abuse are closely associated. Children under five are disproportionately present in families experiencing partner violence, are at greatest risk of child maltreatment compared with older children and make up over 75% of US maltreatment fatalities [11]. Children may be abused directly or may witness violence, but are commonly stressed by living in a violent environment and so their health and wellbeing suffers. Depending on the length of exposure, nature and severity of violence (and factors such as substance misuse and poverty), partner violence during childhood has been associated with increased displays of aggressive behaviour, emotional problems such as depression and/or anxiety, lower levels of social competence and poorer academic functioning. One of the resiliency factors thought to protect a child from the harmful effects of IPV is a positive relationship with an adult, especially a parent [12].

### Depression and partner violence

Victimised women suffer significantly more mental health disorders than non-abused women, particularly higher rates of post-traumatic stress and depression. Depression is strongly associated with IPV among women attending family doctors (GPs) [13]. In a meta-analysis, Golding found that just under half (48%) of all abused women suffered from clinical depression compared with up to 20% among women in the overall community [14]. Dose-response relationships were evident in both severity (the more severe the abuse, the more severe the depression) and recovery (the longer women were away from the abuse, the greater the reduction in their depression) [14].

The prevalence of depression after childbirth in Australia has been estimated to be 16.9% [15]. Marital disharmony and lack of support are among the most consistent associations with women reporting depression after birth [16], although among such women, the proportion experiencing abuse during the pregnancy is unknown. Maternal depression is associated with poorer cognitive, behavioural and emotional outcomes for children [17].

### Primary care professional management and intervention in partner violence

Pregnant and recent mothers in the state of Victoria have access to universal primary health care services in the form of general practice (GP) care during pregnancy, and maternal and child health (MCH) nurse care until their child reaches five years old. Abused women are over-represented in general practice populations [18]. One or two victimised women a week are estimated to attend unsuspecting GPs, but the prevalence in Australian MCH nurse populations is unknown. Pregnancy is a time when abused and isolated women may be allowed to attend their doctor and MCH nurse, so these are important sites for early intervention [19]. Women experience barriers to seeking help including shame and self-blame; and they may feel unready or too fearful to disclose. Direct questioning by health care professionals can facilitate disclosure and if women are met with consistently supportive attitudes, their disclosure may be beneficial, while harm can result if professionals' responses are judgemental or the violence is dismissed and only the symptoms treated [20]. There are many barriers facing primary care professionals in the detection and management of partner violence. In primary care, the two main recommended interventions are to identify and then to refer women and/or their partners to intervention services [20]. However, few rigorous studies of the effectiveness of IPV interventions for women in the community exist and there are currently no rigorous studies the benefit to women of primary health care IPV referrals [3].

### Reducing intimate partner violence: the necessity of complex interventions rigorously evaluated

Intervening to reduce IPV in primary care settings and to improve health outcomes for women abused involves developing a thorough understanding of what is a complex problem and the pathways by which it is caused and sustained. It also requires determining whether and how these pathways are amenable to change and developing an intervention that is both acceptable and feasible to implement. The extent of developmental work needed for designing such complex health interventions has been raised by others [21,22]. The design of the intervention trial described here was conceptualised over a number of years, with piloting, modification and feasibility testing carried out prior to commencement of the main trial. The aim of this paper is to describe fully the development and implementation of MOSAIC and its participants' baseline characteristics.

### An intimate partner violence intervention: developing MOSAIC

MOSAIC (MOthers' Advocates In the Community) was a cluster randomised trial embedded in primary care. It examined if non-professional support from mentor mothers could reduce partner violence and depression and strengthen the health, wellbeing and mother-child bonds of women who are pregnant or have children under five, identified as abused or symptomatic of abuse by their primary health care providers.

### Evidence behind the design for a mentor mother social support intervention

Lack of social support has been associated with mortality and morbidity across a range of health conditions, whilst the presence of support is thought to involve beneficial effects on recovery and disease course, although the mechanisms are often inadequately explained [23]. In particular, good social support has shown to be associated with good mental and physical health outcomes for women, irrespective of the level of violence experienced [24].

We drew on three evidence-based social support strategies in the context of IPV and social disadvantage to develop the MOSAIC mentor mother intervention:

1. Randomised trials of home visiting (by nurses or para-professionals) and peers (non-professional women) among new mothers in disadvantaged communities have reported some success at improving long term maternal and child emotional and physical health outcomes (including reduced rates of child abuse) when the intervention continues well after birth [25,26]. Home-visiting nurse trials are being replicated in Australia and elsewhere [27]. If chronic domestic violence was present and professional staff did not address the issue directly, results for home visiting have been less successful [25].

2. A randomised trial of 10 weeks of domestic violence advocacy provided by trained para-professional female university students for women exiting refuge/shelters found significant positive intervention effects for women's mental health and well being, social support, access to community resources and level of abuse persisting across a two year period, compared with women who had not been supported. The authors called for evaluation of different types of advocates, e.g. formerly battered women or community volunteers [28-30].

3. In a controlled study, McFarlane *et al *tested a mentor mother model with pregnant Hispanic women in Texas clinics [31]. Combining domestic violence advocacy with home visiting or phone calls by trained mentors from the local community, the study sought to improve women's safety and well-being. Giving identified abused women printed information or hospital-based counselling was compared with mentor mother support during pregnancy, but only up to the birth. The reduction in abuse found during pregnancy in the advocacy arm of the trial was not sustained six, 12 and 18 months after birth. The investigators concluded that the design could have been inadequate as the intervention ceased at birth, a time when pressures on both partners increase with the arrival of the baby [31].

## Methods/Design

### Development and piloting the mentor model

Development and piloting of the MOSAIC intervention model and the design of the trial occurred over a period of three years (2001–2003). GP and MCH nurse identification and referral of women; recruitment of mentor mothers and assessment of their acceptability to referred women, and the training program for mentors, were each pilot tested in this development phase.

### Piloting activities and results

• A **scoping **exercise found that volunteer mentor models were feasible with disadvantaged new mothers, but none targeting IPV victims existed.

• **Support for the model **– GPs and MCH services reported finding caring for partner violence victims challenging. Five urban GP divisions (Victorian GP administrative organisations) offered their formal written support to participate, representing over 790 GP practices. Both health care professionals and family violence services supported the proposed mentor model.

• **Feasibility of referral in primary care**. Untrained GPs in 4 practices piloted identification and referral of women using draft referral resources and found resources to be acceptable and referral feasible. GPs in 3/4 practices actually referred. Over 4–6 weeks, 18 women were identified, 11 women were invited to participate and 6 agreed. This indicated that for 30 practices (assuming a non-response by 10/40 practices) over 40 weeks, 5400 women could be identified, 3300 invited and 1800 agree. This suggested a healthy margin to achieve the original sample size of 700.

• **Recruitment and acceptability of mentors **was piloted and found to be feasible, including recruitment of Vietnamese mentors. A mentor training program was developed and trialled with recruited mentor mothers and a small number (10) of women referred by one pilot MCH intervention team were matched with a mentor and supported for 12 months

• The **survey instrument **was developed, piloted and amended in English, and acceptable to the target group. In addition, it was translated into Vietnamese, back translated and then focus group tested with Vietnamese psychologists and bilingual family violence workers.

### The MOSAIC model

#### Mentoring and advocacy

For women identified as abused or at risk of abuse, MOSAIC mentor mothers provided regular contact for up to 12 months. This included:

• Providing non-judgemental listening, support and friendship;

• Maintaining contact (weekly on average) and support through phone calls, home visiting and outings;

• Assistance in developing safety strategies appropriate to women's circumstances;

• Developing a trusting relationship and modelling a sense of hope;

• Providing information and support with parenting; and

• Providing information about, and assisted referral to community services (especially family violence services) and resources for women and their children.

#### Mentor mother recruitment, selection and training

MOSAIC employed either one or two coordinators (depending on the phase of the study) to train, support and supervise mentors. The MOSAIC Coordinators recruited mentor mothers through local newspapers, volunteer networks, flyers and radio. They undertook thorough screening of volunteer and paid mentor mothers [32]. This included (a) an initial inquiry telephone interview, (b) eliciting information through a written application, (c) an intensive panel interview, (d) a police check, (e) three confidential referee checks, and (f) 5 days of paid and compulsory training.

The training was designed to focus on: values and attitude clarification and the importance of being non-judgemental as a mentor mother; an understanding of family violence issues and services, including a strong focus on safety; understanding depression in the context of motherhood; communication skills, especially ways of listening respectfully; effective ways of providing emotional support to vulnerable mothers; the qualities of supportive friendship; the importance of confidentiality; cross-cultural understandings; women's health and self care; and issues of grief and loss. The project training and coordination manual which guided the MOSAIC mentor mother program is available elsewhere [32].

#### Mentor mother coordination and support

MOSAIC Coordinators were responsible for calling or visiting a woman referred to the intervention arm of the study to assess her situation and her preferences for a mentor. The Coordinator was then responsible for approaching a suitable mentor and arranging a match meeting. While a woman was waiting to be matched, a mentor support officer rang the women to provide them with interim telephone support.

A Coordinator accompanied the mentor to meet the woman and facilitate an understanding of the roles and responsibilities, boundaries, and goals (if appropriate) of the new relationship. This included the option for either woman to agree or not to the match. The Coordinator rang both women 24 hours later to ascertain whether each was keen to continue or not. There were a few times when women expressed the desire for another mentor following an introduction and a rematch was then required. Additionally, a mentor might need to leave the program for unavoidable reasons (e.g. moved interstate, or commenced full-time employment) and a rematch was then required.

Mentors were encouraged to contact a Coordinator when visits with women were challenging, required information which the mentor could not provide at the time, or for any other additional support. All mentors were provided with mobile phones for their personal safety and to ensure that mentors bore no financial costs of contacting women or Coordinators.

The Coordinators conducted and documented four monthly reviews with women to see how the relationship with the mentor was going from the woman's point of view. Participants were also able to contact the Coordinator at any time if they felt the need to talk about how their mentor support was going or to seek additional specialist advice about particular circumstances. Coordinators also regularly reviewed fortnightly contact sheets completed by mentors to monitor progress with the participant. This may have required her contacting the mentor if there had not been satisfactory feedback or contact sheets were not submitted, or if the Coordinator judged that there was any concern for the welfare of the mentor or the participant which required contact.

MOSAIC Coordinators organised six to eight weekly meetings for both matched and unmatched mentors to get together to share their experiences of any difficulties and successes. These meetings were important to mentors' involvement in the project and strengthened the support with which they were provided. The structure of the meetings involved a mixture of peer support, support from the Coordinators, information sessions and occasional guest speakers on topics of interest (e.g. new state family violence laws or child protection services).

Finally, MOSAIC also aimed to provide opportunities for growth and leadership among mentor mothers. Some mentors were survivors of violence themselves who wished to give back to their communities and they modelled this to the women for whom they cared. Others were refugees or immigrants wishing to help their communities and build their skills for later employment in the community. MOSAIC training and experience offered mentors a pathway to sustained community engagement and possible further employment.

#### Implementing the intervention in the Vietnamese community

Partner violence in ethnic minority communities is inadequately studied. Ethnic minority women report low satisfaction with maternity care, have poor access to early intervention when abused, and are over-represented in partner homicide data. The study was designed to be inclusive for one ethnic minority community – Vietnamese women, the largest of the ethnic minorities among childbearing women in Victoria. Unfortunately, inclusion of more than one ethnic community was prohibitive, given the considerable extra costs of research that is ethnically inclusive [33]. Other reasons for a focus with the Vietnamese community included: their visibility in crisis and outreach domestic violence services, suggesting poor access to early intervention and a significant burden of abuse [34]; under-representation of Vietnamese GPs in continuing IPV professional development programs; and the existence of previously translated and validated versions of two study instruments in Vietnamese (The Edinburgh Postnatal Depression Scale [35]; and SF 36 [36].

### Study Design

MOSAIC was designed as a cluster-randomised trial in which GP practices and MCH nurse services formed the units of randomisation [37].

### MOSAIC Aims

The primary aims of the trial were:

• to reduce partner violence by 16% among women pregnant and/or with children under five whom GPs or MCH nurses identify as abused or at risk;

• to reduce depression by 16% in women pregnant and/or with children under five whom GPs or MCH nurses identify as abused or at risk; and

• to strengthen the general health and wellbeing and mother-child bonding of abused or at-risk women.

The secondary aims were:

• to strengthen infrastructure support for primary care management of partner violence, by enhancing effective inter-sectoral collaboration between general practice and community-based family violence networks; and

• to enhance primary care case management of family members living with partner violence.

The major hypothesis tested in MOSAIC was that mentor mother support would reduce both partner abuse and depression and the hypothesised pathway for this to occur was the following:

• that by reducing isolation, providing links to community resources and using strength-based support, sustained help from mentor mothers to women who are pregnant and/or have children under five referred by their primary care provider will encourage women to take actions to increase their safety; and

• that any reduction of abuse, together with continued non-professional mentor support, will reduce the level of depression and enhance women's general health and mother-child bonding.

### Recruitment of the clusters: GP practices and maternal and child health teams

Prior to study commencement, support for MOSAIC was obtained from the five General Practice Divisions in the planned catchment area for the study, and permission granted to contact GP practices in the region. General practitioners (GPs) were invited from practices included in the Maternity Affiliate Shared Care List for the Victorian Department of Human Services North West Region, and GPs who were members of the five participating Divisions of General Practice (including all Vietnamese GPs in the region) were also sent letters of invitation. Vietnamese GPs were approached in person by a Vietnamese project officer and invited to participate. MCH nurse teams located in six local government areas in North West Melbourne were informed about MOSAIC and invited to participate.

Formal written invitations included information about: the rationale and design of MOSAIC; the requirement for participation in six hours of professional development addressing IPV identification and management; a commitment to invite and refer women to the study for a required period of time; acceptance of the process of randomisation; a copy of the Memorandum of Understanding regarding participation; and contact details for further information about the trial.

24 GP practices (27 GPs) and six (later increased to eight) MCH teams agreed to participate by returning a signed copy of the Memorandum of Understanding.

### Professional development about intimate partner violence

Randomised trials with abused women are a controversial issue, because of the ethical concern about what would be withheld to women in the comparison arm. In order to provide all women in the study, comparison women included, with an enhanced primary health care service (one in which health care professionals would all have had training to identify and refer women to family violence or other support services), MOSAIC provided an initial full day's IPV training and resources (manuals, referral booklets, pamphlets), to all participating health care professionals. GP and nurse participation in MOSAIC was contingent on taking part in professional development designed to enhance:

• knowledge about the prevalence and sequelae of partner violence, especially in the childbearing years;

• skills in recognising symptoms of victimisation, perpetration and the impact on children;

• sensitive inquiry about abuse

• a supportive response; and

• effective referral of family members to appropriate services.

The GP training program was informed by a new curriculum and international consensus guidelines developed by two MOSAIC investigators (KH and AT) to provide GPs with identification and management strategies for all members of the family (victims, perpetrators and children) experiencing partner violence [38]. Professional development points were awarded toward annual accreditation for participating GPs. Interactive adult learning strategies and values-identification were key features of the training program [39]. The program consisted of six hours of:

▪ readings and formal lectures about prevalence and sequelae;

▪ hearing the direct experiences of victim and ex-perpetrator consumer consultants;

▪ small-group discussion about case studies; and '

▪ fish bowl' practice consultations with simulated patients.

Family violence service providers for victims and perpetrators in the region explained their services to GPs. Similarly sessions for MCH nurse teams also involved six hours of interactive learning strategies, readings, presentations, videos, small-group discussion and additional resources for self-learning. While the focus of MCH nurse training was mainly on the needs of abused women, nurses were also interested to explore their role with abusive partners. Following the training, general practices and MCH nurse teams were then randomised.

### Randomisation

GP practices and MCHN teams were randomised at separate public meetings following completion of the professional development programs. For GPs, names (stratified by practice size – one GP or more than one GP participating) were concealed in opaque envelopes and randomly selected by someone outside the study. Similarly names of the MCHN teams were concealed in opaque envelopes, with teams stratified by the number of births in the local government area and the selection made by an invited guest from outside the project or research team. GPs and MCH nurse Team Leaders were present to ensure the fairness of the process and to check the contents of the envelopes prior to their being sealed.

### Blinding

MOSAIC is a pragmatic intervention study. Due to cluster randomisation, it was not possible to blind the health care providers to their status when different processes were required in order to introduce women to the study and refer them to the research team in different arms [21]. Similarly, research staff were not blind to participant status as differing levels of information and follow-up were required for participants in the different arms of the study.

### Ethical and safety issues

MOSAIC received ethics approval from the Human Ethics Committee at La Trobe University (03–76) and the University of Melbourne (030441). Safety and confidentiality are priority ethical issues for any study involving victims of IPV [40,41]. MOSAIC safety protocols involved agreed processes to be followed to enhance the safety of participants, staff and mentors, including: assessment of safety prior to visiting women; women's expressed choice of safe location; what to do in any situation where the perpetrator may answer the phone, come home unexpectedly when a mentor or staff member is visiting, or contact or follow MOSAIC staff members. In all cases the protocols aimed to ensure that project activities did not jeopardise participant safety. Additional protocols covered project responsibilities and processes regarding child abuse if this was identified and suicidal ideation if this was reported (see ref. 32 Appendices). All staff had mobile phones and were "buddied" with another staff member to whom they told their leaving time and location and to whom they were asked to report when interviews were complete. Coordinators and mentors were also provided with mobile phones and were trained in safety procedures and making safety plans. As indicated above, we gave all participating health care professionals in either arm extensive IPV training and referral resources so that, we hypothesised, all women in the study would be cared for by well-trained and resourced providers.

### Recruitment and consenting of participants

The participating GPs and MCH nurses were asked to fax, phone or email contact details of women they had identified as eligible (abused or psychosocially distressed and indicative of abuse) and who were willing to participate in a study of emotional support for mothers experiencing difficulties. Research staff then contacted women to arrange for a safe time and place to provide women with more explicit information, negotiate consent, administer a baseline survey and give all women a list of local and statewide resources for mothers, including information about family violence services. In the intervention arm, the research officer then contacted the Coordinator and arranged a suitable time for her to visit. Women whose English rendered them unable to give informed consent (whose language was not Vietnamese) or those with unmanaged serious mental health issues were excluded.

### Ongoing health care provider support

A number of strategies were employed to ensure effective liaison between MOSAIC staff and GP practices and MCH teams. These included: project updates sent via a regular project newsletter; ongoing provision of appropriate posters and pamphlets in relevant community languages as well as in English when requested; offering opportunities for further upskilling following interim process evaluation; facilitating opportunities to meet with local family violence and related services; and providing timely feedback about any referrals made.

### Sample size considerations

With 80% power and 95% confidence, MOSAIC aimed to detect a difference of 16% in abuse or depression a year after recruitment. This was originally estimated to require 165 women in each trial arm with individual randomisation. This reduction was thought to be attainable from a level of any prevalence value for either IPV or depression between 30% and 70% in the control group of women already identified by their GPs as abused or at risk. With cluster randomisation, assuming an intra-class correlation of 0.02 (previously found in a GP partner violence study [18]), the sample size required increased to 190 in each arm. Given the mobility of this vulnerable, sometimes fearful population an expected attrition and loss to follow up of as much as 45% could be expected, requiring 350 to be recruited in each arm. Piloting data indicated that 40 weeks would be required for referral and recruitment of this required number of participants.

By May 2007 (well beyond the 40 week period), both referral and recruitment had been slower than expected and the ratio of intervention to comparison for both those referred and recruited was approximately 2:1. At this stage 131 women in the intervention arm and 71 women in the comparison arm had been referred and of these 63% had been recruited. It was decided to extend the recruitment period for another 7 months, and include one additional MCH nurse team in each arm of the trial.

The estimated sample size was recalculated and we then estimated that, for simple random sampling, with 118 in the intervention arm and 64 in the comparison arm, the study would have 80% power and 95% confidence of being able to detect a difference between arms of 22% for IPV, 18% for depression and two units in the mental component score (MCS) of the SF36. Allowing for the overall 37% attrition rate seen to that time and the 10% inflation factor for the cluster sampling, we hoped to achieve total referrals of 206 women (+75) in the intervention arm and 112 (+41) in the comparison arm to be referred in the extra time period.

### Data collection

Baseline data were collected on recruitment and outcome evaluation data were collected twelve months later. We repeated the two primary outcome measures after twelve months of mentoring in the few instances where there was a delay of three months or more in a participant being allocated a mentor. Any woman who was offered a mentor received a supplementary questionnaire about her experience of mentoring.

#### ◦ Outcome evaluation

Abuse status was measured using the Composite Abuse Scale (CAS), a comprehensive, well-tested and validated screening tool which discriminates between differing forms of abuse (severe combined abuse, physical abuse only, emotional abuse and harassment) [42]. Depression was measured using the Edinburgh Postnatal Depression Scale (EPDS) [43], which has been validated with mothers (and fathers) outside the immediate postpartum period [44,45]. Women's general health status was measured using the SF36 [36]. The mother-child relationship was measured using the Parenting Stress Index (PSI) Short Form, which can be used for parents of children as young as one month [46] and has been used previously with women experiencing abuse. The PSI Short Form (SF), has three subscales derived from and well validated against the long form: – Parental Distress, Parent-Child Dysfunctional Interaction (P-CDI) and Difficult Child. The P-CDI subscale elicits threat to the parent-child relationship [46]. Use of and satisfaction with GP and MCH nurse care was measured via participant self-report.

#### ◦ Process and impact evaluation

Multiple methods have been used to evaluate the study's processes during implementation and following the completion of the study [47]. One year into the two year referral period, we conducted telephone interviews with (74/91 81%) MCH nurses and (13/27, 48%) GPs to assess their views of their training and support in order to respond with further strategies to encourage enhanced identification and referral of women. After the referral period was complete, surveys of participating MCH nurses and GPs sought their views on the impact of MOSAIC on their practice and the nature of the intervention. In-depth interviews with 30% of mentored women (n = 36 including 11 Vietnamese women) and 26% of mentors (n = 18 including 4 Vietnamese mentors) explored their overall experiences of mentoring and being mentored.

#### ◦ Economic evaluation

MOSAIC involved a significant investment of economic resources (time and money) from health professionals, training providers, mentors and women at risk. Participation in MOSAIC might also lead to a change in use of health care and other services that could increase or reduce the total provision of services to women at risk. In recognition of this investment, MOSAIC included economic evaluation to assess the outcomes achieved relative to the cost of resources required to achieve them. With multiple outcomes of interest, the economic evaluation took the form of a cost-consequences analysis where the difference in costs experienced between the two trial arms is compared to the difference in all outcomes included in outcome evaluation.

### Data analysis and reporting

The primary analysis will be by 'intention to treat' (allocation): first, descriptive statistics of the characteristics of the Intervention and Comparison groups at recruitment have been calculated to check that cluster randomisation resulted in similar groups (see Tables 1 &2 below). With the main analysis, a comparison of the primary outcomes pre – and post intervention is to be undertaken. These are depression (mean EPDS score and the proportion with a 'probable depression' score (>12)), the proportion experiencing no (0) low levels (CAS 3–6) or high levels of abuse (CAS → 7) on the Composite Abuse Scale), the mean score on the short form of the Parenting Stress Index and the mean subscale score on parent-child interaction. All analyses will be carried out using STATA 10 [48], adjusting for clustering by practice/nurse team. As there is an imbalance in one major prognostic factors despite randomisation, multivariable logistic regression will be used to take this into account. Although numbers of participating Vietnamese women will be too few to undertake adequately powered sub-group analyses, process and outcome data will provide useful information about the acceptability of the intervention in this ethnic community and identify any major trend towards differential outcomes in this group.

**Table 1:** Socio-demographic characteristics of participants in both arms of the MOSAIC study

**All respondents**				
	**Intervention**		**Comparison**	
	n	*%*	n	*%*
**Total**	**114**	***100***	**60**	***100***
***Age group***				
<25	15	***13***	9	***15***
25–29	24	***21***	10	***17***
30–34	34	***30***	22	***37***
40–50	14	***12***	6	***10***
Missing	3	***3***	0	***0***
***Marital status***				
Married	35	***31***	15	***25***
Living with de facto partner	21	***18***	15	***25***
Not living with partner	10	***9***	8	***13***
Divorced or separated	29	***25***	13	***22***
Single	18	***16***	8	***13***
Missing	1	***1***	1	***2***
***Numbers of children***				
1	48	***42***	29	***48***
2	39	***34***	15	***25***
3	20	***18***	9	***15***
4+	6	***5***	7	***12***
Missing	1	***1***	0	***0***
***Level of education***				
Degree	18	***16***	11	***18***
Diploma	25	***22***	9	***15***
***Apprenticeship or traineeship***	14	***12***	9	***15***
Year 12	28	***25***	10	***17***
Less than year 12	29	***25***	21	***35***
***Health care card***				
Yes	86	***75***	42	***70***
No	27	***24***	18	***30***
Missing	1	***1***	0	***0***
***Income source***				
Wages or salary	41	***36***	25	***42***
Pension or benefit	70	***61***	34	***57***
Missing	3	***3***	1	***2***
***Country of birth***				
Australia	76	***67***	37	***62***
Vietnam	19	***17***	4	***7***
Other	19	***17***	19	***32***
***Australian residents***				
Yes	111	***97***	54	***90***
No	3	***3***	6	***10***
***Years Australian residency***				
Australian	76	***67***	37	***62***
Less than five years	9	***8***	7	***12***
5–14 yrs	9	***8***	9	***15***
14–43 yrs	16	***14***	6	***10***
Missing	4	***4***	1	***2***

**Table 2:** Comparison of abuse and depression status by arm

	**Intervention, n = 114**		**Comparison, n = 60**	
**IPV status**	n	%	n	%
0	9	*8*	6	*10*
3–6	11	*10*	2	*3*
**≥ 7**	93	***82***	48	***80***
Missing	1	*1*	4	*7*
				
**When partner last inflicted abuse**				
***Abused in last 12 months****(n = 121)*				
0	1	*1*	0	*0*
3–6	3	*4*	1	*2*
**≥ 7**	76	***95***	39	***95***
Missing	0	*0*	1	*2*
***Abused>12 months ago ****(n = 33)*				
0	4	*20*	3	*23*
3–6	*3*	*15*	0	*0*
**≥ 7**	13	***65***	8	***62***
Missing	*0*	*0*	2	*15*
***A partner has never abused me****(n = 15)*				
0	4	*36*	2	*50*
3–6	2	*18*	1	*25*
**≥ 7**	4	*36*	0	*0*
Missing	1	*9*	1	*25*
				
**Depressed (EPDS ≥ 13)**				
No	32	*28*	26	*43*
Yes	81	***71***	33	***55***
Missing	1		1	

In reporting the findings of the trial, the CONSORT guidelines will be followed from the point of recruitment. Discussions with GPs and MCH nurses have shown it is not possible to identify reliably, through existing systems, women attending who are pregnant or come with a child under five, so that a count of the potentially eligible source population will not be available [49].

For qualitative data, sampling of mentors and women has been conducted to achieve maximum richness and diversity of experience [50]. In addition, coding frameworks will be developed and cross-coding implemented to check consistency, following which thematic analyses examining the views and experiences of matched pairs of participants and mentors and those of MCH nurses and GPs will be undertaken.

### Sample description

Table 1 below describes participant characteristics in both arms of the trial. There are very few differences in the socio-economic profiles of participants in the two arms of the study. A slightly higher proportion of women in the comparison arm have less than 12 years of education. However, both groups have a high percentage of women with indicators of disadvantage. Over 70% in both arms have a health care card (i.e. government coverage of health care costs for those with inadequate income), over half are dependent on a pension or benefit (e.g. single-parent support), over half have only completed either apprenticeship level, year 12 education or below and while most are Australian citizens, over a third are born overseas. Very few Vietnamese women (n = 4) were referred in the comparison arm.

Table 2 describes the distribution of scores at baseline on primary outcome measures, namely levels of abuse and depression. GPs and nurses identified abused and depressed women to MOSAIC, but at very low levels over the extended two year period. The proportion of women abused (CAS ≥ 7) is comparable between arms (≥ 80%), and the proportion of women abused among those victimised in the previous 12 months (the majority) is equal in both arms (95%). The proportion of women depressed (EPDS ≥ 13) is higher in the intervention arm than in the comparison arm, with a difference in mean baseline EPDS score (15.4 in the intervention arm, 13.0 in the comparison arm).

## Discussion

MOSAIC experienced much lower rates of referral to the project than had been suggested by the experiences in the pilot, with the added problem of proportionally fewer referrals in the comparison arm of the trial. We discuss the implications below.

When it became apparent that referrals were low, five strategies were implemented. First, all participating MCH teams and GP practices were asked, and agreed, to extend the referral period from 40 weeks to 24 months. Second, two further MCH teams were invited, and agreed, to participate in MOSAIC. Third, we developed a specific poster in conjunction with one of the referring MCH teams, for use in waiting rooms. The poster "You don't have to tell us, but you can" let women know that their MCH nurse or GP had participated in a professional development program about supporting mothers experiencing IPV and was designed to encourage women to talk with providers if they wished. Fourth, feedback gleaned from process evaluation interviews with MCH nurses and GPs about the barriers to referral was used to tailor ongoing development sessions with the participating doctors and nurses, focusing very specifically on recognising the signs and symptoms of IPV and on supportive ways of asking women about their relationships with their partners that might appropriately elicit disclosure. Fifth, the importance of referrals in the comparison arm was emphasised in subsequent project newsletters and in discussion with nurse teams and GPs. Positive feedback about participation in the study from the women already referred to MOSAIC from the comparison arm, was also communicated to all nurses and GPs. Despite these strategies, final referrals to the trial indicated a reluctance to refer in the comparison arm.

It might be argued that reasons for the low referral to MOSAIC were associated with the study. However when we compared MOSAIC referral rates over the same two-year period to MCH nurse referral rates for IPV which they are required to provide to the state health department, they were broadly similar and in two areas, more frequent to MOSAIC (because, we were told, we responded more quickly than community based services).

There is almost no evaluation of the effectiveness of IPV training and support to health care professionals and there remains an urgent need to find methods which result in beneficial behaviour change [51]. All eight MCH nurse teams are now participating in a further trial with the authors to evaluate a new model of IPV training and support, demonstrating a recognised need and their willingness to improve their capacity to care for abused women and their families.

### The potential contribution of MOSAIC

MOSAIC is the first randomised trial of mentor mother support for abused women anywhere in the world and the first randomised trial of an IPV intervention in Australia. There are very few interventions in primary health care which address intimate partner violence; and of these, even fewer which have been evaluated with a rigorous design to estimate the benefit for women and children [52,53]. The MOSAIC study drew from two pioneering IPV support studies [28,54] and developed a new model to offer and evaluate twelve months mentor mother support to women attending primary health care services during the challenging period both before and after a child is born. MOSAIC will contribute to evidence of effectiveness (and lack of harm) for women and their children.

There is also an ongoing debate about the relative value of health care professional (home visiting nurses) compared with non-professional support to improve the health and wellbeing of abused women and children. MOSAIC will contribute to examining and exploring the contribution mentor mothers can make in addition to professional support and how women perceive the benefits or harms of such relationships.

Asking all health care professionals to screen for women experiencing partner violence in health care settings is a continuing and controversial policy issue. Educational interventions in health care addressing IPV which result in sustained behaviour change are very few and some undesirable outcomes have been reported [55-57]. Through its new curriculum, and process and impact evaluation, MOSAIC hopes to contribute to further learnings about improving education and support to health professionals caring for women suffering from intimate partner violence.

## Abbreviations

IPV: (intimate partner violence); GP: (general practitioner); MCHN: (maternal and child health nurse).

## Competing interests

The authors declare that they have no competing interests.

## Authors' contributions

AT had major responsibility for the design and conduct of the MOSAIC study, co-developed and delivered the GP training and drafted and revised the manuscript. RS provided substantial input to the design and implementation of MOSAIC and drafting and revising of the manuscript. KH contributed to the design of MOSAIC, the development and delivery of GP training and drafting and revising the manuscript. JL contributed to the design of MOSAIC. LW has provided statistical design advice and analysis and contributed to the draft manuscript. LG designed the economic analysis and contributed to the draft manuscript. All authors approved the final manuscript.

## Authors' information

Angela J Taft MPH PhD a.taft@latrobe.edu.au

Senior Research Fellow, Mother and Child Health Research, La Trobe University, Australia

Rhonda Small PhD

Associate Professor and Acting Director, Mother and Child Health Research, La Trobe University, Australia

Kelsey L Hegarty MBBS PhD, Associate Professor, Department of General Practice, University of Melbourne, Australia

Judith Lumley MBBS MA PhD

Emeritus Professor, and former Director, Mother and Child Health Research, La Trobe University, Australia

Lyndsey F Watson M Sc PhD, Senior Research Fellow and Statistician, Mother and Child Health Research, La Trobe University, Australia

Lisa Gold MSc, Senior Research Fellow, Deakin Health Economics, Deakin University, Melbourne, Australia

## Pre-publication history

The pre-publication history for this paper can be accessed here:


